# New Insight into Drugs to Alleviate Atopic March via Network Pharmacology-Based Analysis

**DOI:** 10.3390/cimb44050153

**Published:** 2022-05-18

**Authors:** Ki-Kwang Oh, Md. Adnan, Dong-Ha Cho

**Affiliations:** Department of Bio-Health Convergence, College of Biomedical Science, Kangwon National University, Chuncheon 24341, Korea; nivirna07@kangwon.ac.kr (K.-K.O.); mdadnan1991.pharma@gmail.com (M.A.)

**Keywords:** atopic dermatitis, allergic rhinitis, allergic asthma, atopic march, protein–protein interaction, molecular docking test

## Abstract

In the present study, a subject of atopic dermatitis (AD) is exposed progressively to allergic rhinitis (AR) and asthma (AS), which is defined as atopic march (AM). However, both the targets and compounds against AM are still largely unknown. Hence, we investigated the overlapping targets related directly to the occurrence and development of AD, AR, and AS through public databases (DisGeNET, and OMIM). The final overlapping targets were considered as key targets of AM, which were visualized by a Venn diagram. The protein–protein interaction (PPI) network was constructed using R package software. We retrieved the association between targets and ligands via scientific journals, and the ligands were filtered by physicochemical properties. Lastly, we performed a molecular docking test (MDT) to identify the significant ligand on each target. A total of 229 overlapping targets were considered as AM causal elements, and 210 out of them were interconnected with each other. We adopted 65 targets representing the top 30% highest in degree centrality among 210 targets. Then, we obtained 20 targets representing the top 30% greatest in betweenness centrality among 65 targets. The network analysis unveiled key targets against AM, and the MDT confirmed the affinity between significant compounds and targets. In this study, we described the significance of the eight uppermost targets (CCL2, CTLA4, CXCL8, ICAM1, IL10, IL17A, IL1B, and IL2) and eight ligands (Bindarit, CTLA-4 inhibitor, Danirixin, A-205804, AX-24 HCl, Y-320, T-5224, and Apilimod) against AM, providing a scientific basis for further experiments.

## 1. Introduction

Atopic lesions, including atopic dermatitis (AD), allergic rhinitis (AR), and allergic asthma (AA), have seen elevated incidence rates for some decades, impacting around 30% of the world population [1]. The atopic march (AM) occurs in a time-based sequence: from AD in infancy to AR and AA in childhood, which is developed from the skin to the respiratory tract [2]. Firstly, AD is a chronic autoimmune skin disorder caused by increases in serum Immunoglobulin E (IgE) levels and hypersensitivity to a diverse array of allergens, including food and bacteria [3,4]. AD is an initial step in the development of AM, and IgE is a key biomarker of allergic inflammation [5].

The first line drug of AD is topical corticosteroids such as Pimecrolimus and Tacrolimus, the mechanism of which is to block the T cell’s activation to dampen immune responses [6,7].

Secondly, AR is an inflammatory disease of the nasal mucous membrane mediated by IgE [8]. The first line drug of AR is H1 antihistamine drugs such as cetirizine, levocetirizine, desloratadine, loratadine, and fexofenadine [9,10]. Thirdly, AA is a final step of AM, and the recommended drugs to alleviate AM are inhaled corticosteroids which suppress airway inflammation [11,12]. Collectively, a key symptom of AM is the inflammatory response, and the optimal strategy to relieve AM is to elucidate overlapping targets from AD, AR, and AA.

The analysis of protein–protein interaction (PPI) networks for drug repositioning is a significant approach, providing clues about causal relationships between targets that carry out certain functions [13]. Therefore, this research has focused on the topological properties of PPI networks to uncover the relatively important nodes among the overlapping targets from AD, AR, and AA.

## 2. Hypothesis

The targets on AD, AR, and AA were retrieved through DisGeNET, OMIM, and the literature, suggesting that the overlapping targets from the three diseases were considered as AM-related targets. We hypothesize that targets with the 30% greatest betweenness centrality (BC) from targets with the top 30% highest degree centrality (DC) values [14] in the overlapping targets are core targets to alleviate AM, thereby identifying the most stably binding ligands on them via molecular docking test (MDT).

## 3. Methods

The targets associated with AD, AR, and AA were acquired through DisGeNET (https://www.disgenet.org/) (accessed on 2 December 2021), and OMIM (https://www.omim.org/) (accessed on 3 December 2021), which are therapeutic relevant components. The overlapping targets from the three diseases (AD, AR, and AA) were considered as core targets of AM. The core targets were analyzed using the STRING database (https://string-db.org/) (accessed on 4 December 2021). We performed the topological analysis of AM on a PPI network. The workflow is as below.

Step 1: Identifying of targets (AD, AR, and AA) from DisGeNET and OMIM.
Step 2: Identifying of overlapping targets (AD, AR, and AA) via a Venn diagram.
Step 3: The overlapping targets were screened on the top 30% by degree centrality (DC), and the PPI network was constructed on RPackage software.
Step 4: After constructing the PPI network of the top 30% DC, the core targets were identified, and the PPI network of the top 30% by betweenness centrality (BC) in the top 30% DC were constructed on RPackage software.
Step 5: Suggesting that targets with the highest DC in the top 30% BC targets were considered as the promising targets against AM.
Step 6: The retrieval of ligands from Selleckchem.com (https://www.selleckchem.com/) (accessed on 24 April 2022).
Step 7: The first screening of ligands based on TPSA <140Å^2^ or Lipinski’s rule.
Step 8: The second screening of Step 7 ligands according to the docking score (<−6.0 kcal/mol) or the lowest binding energy (the highest negative value) of each target.
Step 9: The identified ligands were converted to .sdf format from PubChem into .pdb format utilizing Pymol, and the ligands were converted into .pdbqt format via AutoDock. Then, the PDB ID of targets were obtained via RCSB PDB (https://www.rcsb.org/) (accessed on 7 December 2021). The AutoDockTools-1.5.6 software was utilized to evaluate the affinity of the promising targets and ligands. The ligands were docked with targets using autodock4 by arranging 4 energy ranges and 8 exhaustiveness as default to identify 10 different poses of ligands [15].

The docking site was set in a cubic box on the center: CCL2 (x = 61.620, y = 0.356, z = 0.582), CTLA4 (x = 22.471, y = 17.954, z = −35.403), CXCL8 (x = 0.977, y = −7.328, z = 2.055), ICAM1 (x = −5.082, y = −12.607, z = 35.679), IL10 (x = 17.574, y = 49.569, z = 27.605), IL17A (x = 77.528, y = −4.484, z = -51.759), IL1B (x = 19.495, y = 2.991, z = 73.516), IL2 (x = 6.750, y = 25.891, z = 14.189)

**Figure 1:**
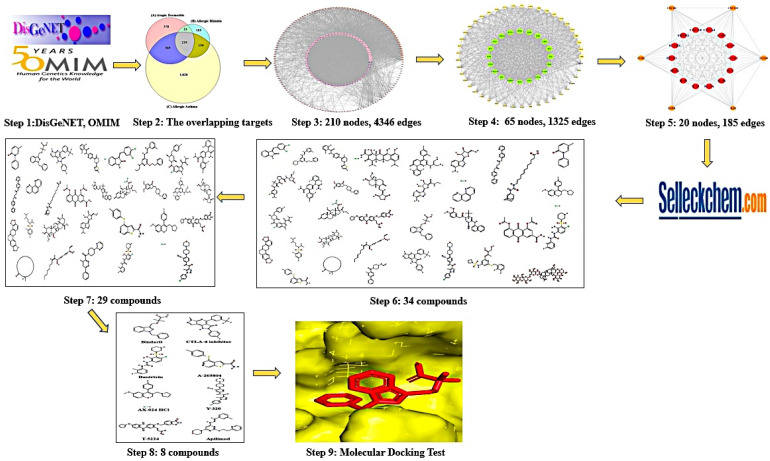
The workflow of this study.

The grid box size was set to 40 Å × 40 Å × 40 Å. The LigPlot+ v.2.2 (https://www.ebi.ac.uk/thornton-srv/software/LigPlus/) (accessed on 8 December 2021) was used to parameterize 2D binding interactions [16]. The adopted ligands on key targets were regarded as key compounds against AM. The methodology workflow is represented in Figure 1.

## 4. Results

**Figure 2:**
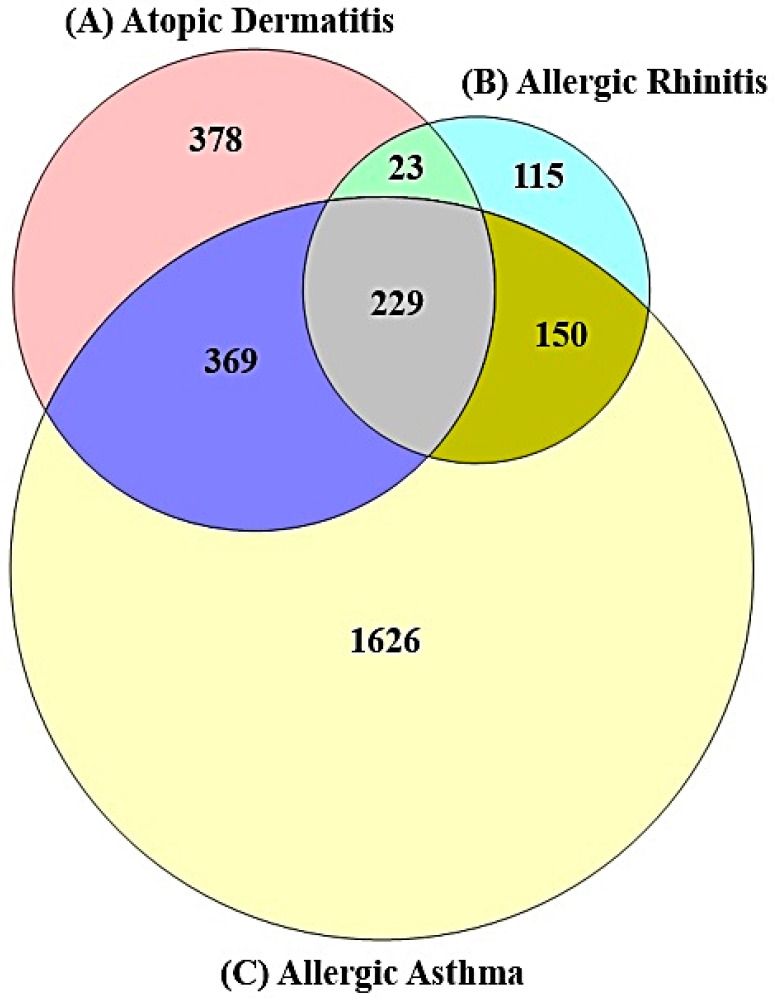
A total of 229 targets related to Atopic March (AM). (**A**) A total of 999 targets related to Atopic Dermatitis (AD). (**B**) A total of 517 targets related to Allergic Rhinitis (AR). (**C**) A total of 2374 targets related to Allergic Asthma (AA).

**Figure 3:**
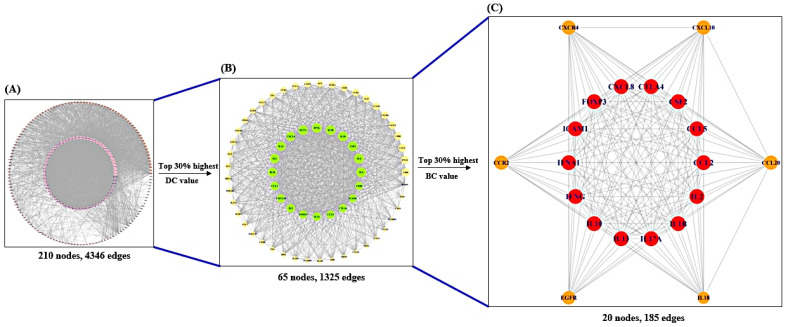
Topological analysis of AM on the PPI network. (**A**) The PPI network of AM targets (210 nodes, 4346 edges). (**B**) The PPI network of significant targets filtered from A (65 nodes, 1325 edges). (**C**) The PPI network of the uppermost targets against AM filtered from B (20 nodes, 185 edges).

A total of 999 (AD), 517 (AR), and 2374 targets (AA) were retrieved by DisGeNET (https://www.disgenet.org/) (accessed on 4 December 2021) and OMIM (accessed on 4 December 2021) (https://www.omim.org/), of which there were 229 overlapping targets (AM) (Figure 2). The target information is profiled in Supplementary Table S1. The 229 targets were considered as core targets against AM, which was comprised of 210 nodes and 4346 edges (Figure 3A) (Supplementary Table S2), and the subnetwork was obtained via selecting the top 30% by degree centrality (DC), containing 65 nodes and 1325 edges (Figure 3B) (Supplementary Table S3). After filtering the top 30% of the subnetwork by betweenness centrality (BC), the key network was assembled, including 20 nodes and 185 edges (Figure 3C) (Supplementary Table S4).

**Figure 4:**
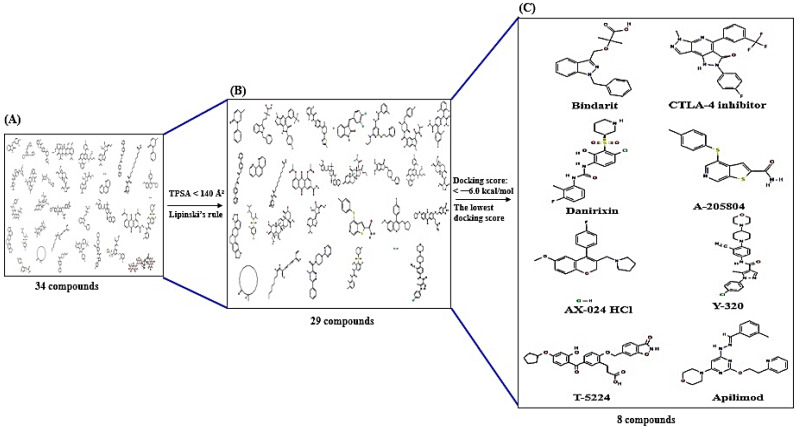
The filtering processing of significant compounds against AM. (**A**) A total of 34 compounds were related to the uppermost 8 targets. (**B**) A total of 29 compounds were filtered from A (TPSA <140 Å^2^ or Lipinski’s rule). (**C**) A total of the uppermost 8 compounds were filtered from B (docking score < −6.0 kcal/mol or the lowest docking score).

Based on the top 30% of the subnetwork by betweenness centrality (BC), 14 out of 20 targets had the same degree centrality (19) (Table 1), which were determined as crucial targets in the PPI network. Then, 8 out of 14 targets were reported with ligands, and 6 other targets had no reported ligands. A total of 8 targets were associated with the 34 ligands (Table 2). A total of 34 ligands related directly to the uppermost 8 targets were retrieved via chemical supplies websites and literature (Figure 4A). The 34 ligands were filtered into 29 ligands with TPSA < 140 Å^2^ or Lipinski’s rule (Figure 4B). MDT was performed on the 29 filtered ligands to select the most stable binding ligands for each target, as a consequence revealing that 8 ligands were critical compounds (Figure 4C).

**Figure 5:**
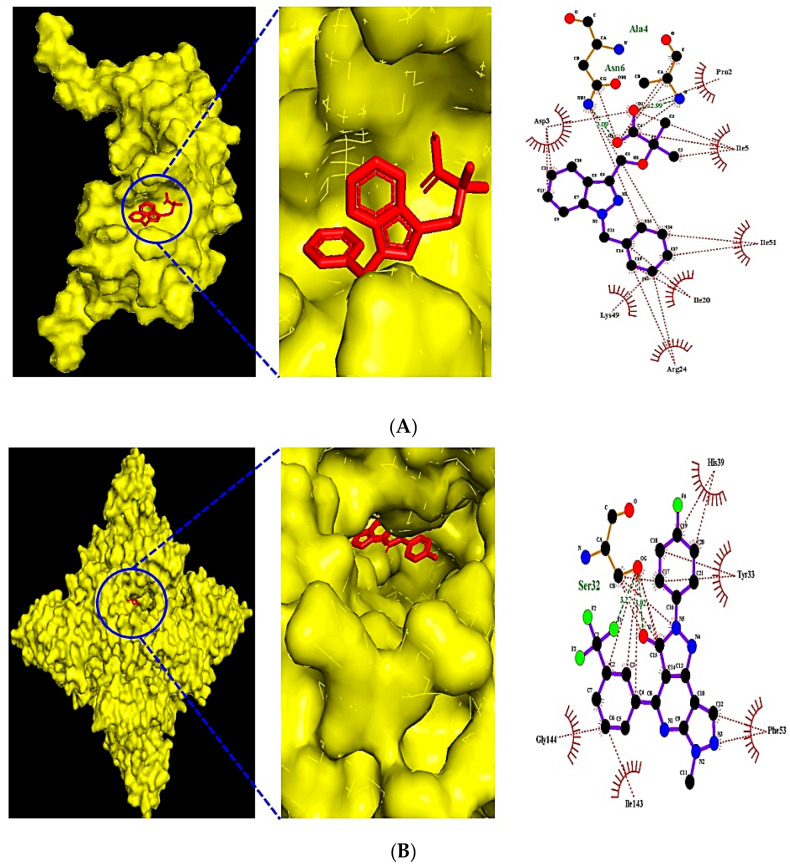

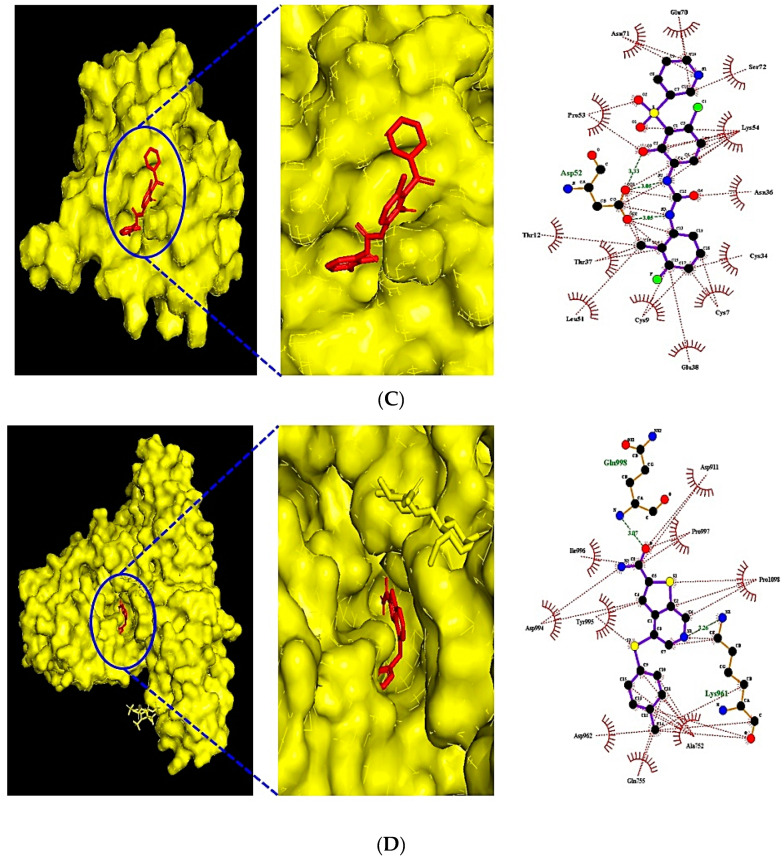

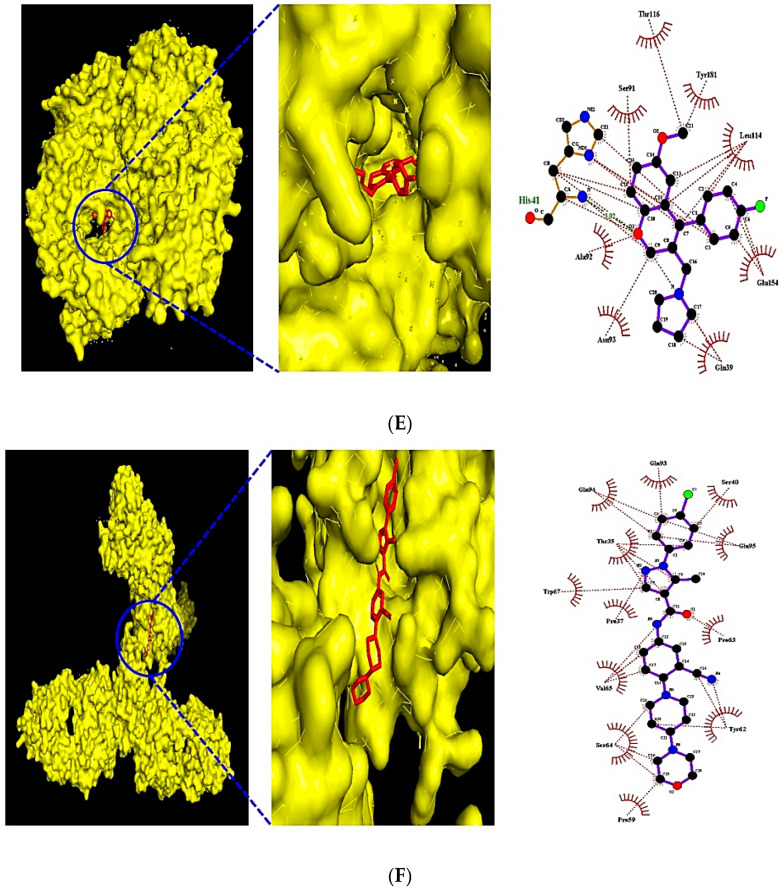

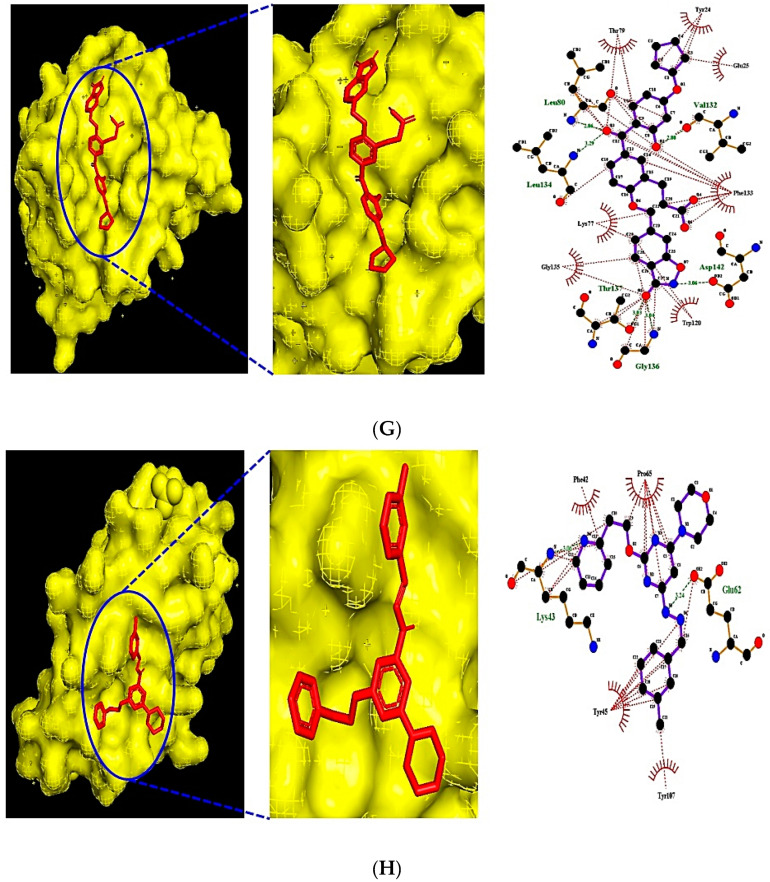
The molecular docking figure of the core eight ligands on key eight targets (**A**) CCL2 (PDB ID: 1DON)—Bindarit (PubChem ID: 71354). (**B**) CTLA-4 (PDB ID: 5XJ3)—CTLA-4 inhibitor (PubChem ID: 101136468). (**C**) CXCL8 (PDB ID: 2IL8)—Danirixin (PubChem ID: 24780598). (**D**) ICAM1 (PDB ID: 5MZA)—A-205804 (PubChem ID: 9839311). (**E**) IL10 (PDB ID: 1LK3)—AX-024 HCl (PubChem ID: 129909862). (**F**) IL17A (PDB ID: 2VXS)—Y-320 (PubChem ID: 22227931). (**G**) IL1B (PDB ID: 1HIB)—T-5224 (PubChem ID: 23646877). (**H**) IL2 (PDB ID: 1M47)—Apilimod (PubChem ID: 10173277).

The MDT of eight ligands demonstrated that (1) Bindarit (PubChem ID: 71354) had the greatest affinity of -7.2 kcal/mol on CCL2 (PDB ID: 1DON) (Figure 5A), (2) CTLA4 inhibitor (PubChem ID: 101136468) had the highest affinity of −7.9 kcal/mol on CTLA4 (PDB ID: 5XJ3) (Figure 5B), (3) Danirixin (PubChem ID: 24780598) had the greatest affinity of −7.0 kcal/mol on CXCL8 (PDB ID: 2IL8) (Figure 5C), (4) A-205804 (PubChem ID: 9839311) had the highest affinity of -8.0 kcal/mol on ICAM1 (PDB ID: 5MZA) (Figure 5D). Most notably, Anemoside B4 (PubChem ID: 71307558) had the greatest affinity of −13.5 kcal/mol on IL10 (PDB ID: 1LK3), but it was violated on three (Hydrogen bond acceptor <10; Hydrogen bond donor ≤ 5; and TPSA <140 Å^2^) by Lipinski’s rule. Hence, (5) AX-024 HCl (PubChem ID: 129909862), with −7.5 kcal/mol on IL10 (PDB ID: 1LK3), was ligand with the most potential on IL10 (PDB ID: 1LK3) (Figure 5E). (6) Y-320 (PubChem ID: 22227931) had the greatest affinity of −7.7 kcal/mol on IL17A (PDB ID: 2VXS) (Figure 5F). (7) T-5224 (PubChem ID: 23626877) had the highest affinity of −8.0 kcal/mol on IL1B (PDB ID: 1HIB) (Figure 5G). (8) Apilimod (PubChem ID: 10173277) had the greatest affinity of −6.2 kcal/mol on IL2 (PDB ID: 1M47) (Figure 5H). The MDT confirmed the stable binding between eight core ligands as well as eight targets (Table 3).

## 5. Discussion

As a matter of fact, targets with a high betweenness centrality (BC) (the measurement of the shortest route between other targets) are the most significant element to be associated with vital multiple therapeutic utilization and then are a noteworthy value to be repositioned [13]. There is an observational result that the eight core targets (CCL2, CTLA4, CXCL8, ICAM1, IL10, IL17A, IL1B, and IL2) overlapped among AD, AR, and AA were the therapeutic targets with the most potential against AM. In addition, the compounds–targets relationship suggested that the therapeutic effect of AM was directly associated with eight ligands, including three benzenoids (Danirixin, Y-320, T-5224), two heterocyclic compounds (Bindarit, CTLA-4 inhibitor), one organosulfur (A-205804), one organic nitrogen compound (Apilimod), and one phenylpropanoid and polyketide (AX-024 HCl). It was reported that benzenoids isolated from *Antrodia cinnamomea* have a potent anti-inflammatory effects on AD, AR, and AA [17,18]. This implies that the benzenoids classification might be a good ligand to alleviate AM. Investigations into the treatment of heterocyclic compounds have revealed that the compounds are useful for the alleviation of inflammatory and allergic disorders including AD, AR, and AA [19]. Study indicated that organosulfur compounds are inflammatory mediators to dampen interleukin (IL)1B and IL17, particularly, AD, AR, and AA [20,21,22,23]. It was reported that nitrogen-containing compounds such as Apilimod are potent selective inhibitors for AD, AR, and AA [24,25,26]. A report indicated that plant-derived phenylpropanoids (PPPs) are anti-inflammatory agents to modulate underlying inflammatory reactions in vitro or in vivo [27].

CCL2 (C-C motif chemokine ligand 2) is the initial chemokine stimulated by itching and pain symptoms on keratinocytes, fibroblasts, and endothelial cells’ infiltered monocytes and lymphocytes [28]. The overexpression of CCL2 is a trigger to respond to allergies and asthma [29]. It could be thus speculated that the inhibition of CCL2 might be a good strategy for the treatment of AM. Study shows that miR-155 as a CTLA-4 inhibitor has inhibitory effects on AD that could potentially disturb the development of AM [30]. Thus, a CTLA-T inhibitor might be a promising target against AM. There is an observational report suggesting that AD, AR, and AA increased the expression level of CXCL8 in responding to innate immunity [31,32,33]. A report suggested that the intercellular adhesion molecule-1 (ICAM1) is upregulated in AD, AR, and AA, indicating that the ICAM1 is associated directly with AM [34,35,36]. IL10 is multiple-functional cytokine during allergic symptoms and a significant element in the progression of severe allergic diseases, including AD, AR, and AA [37,38,39]. IL17A plays a vital role in the occurrence and development of allergic diseases, particularly, AD, AR, and AA [40,41]. IL1B is a proinflammatory agent, mediated by a rise in the expression of ICAM-1, and thereby is related directly to the inflammatory responses such as AD, AR, and AA [42,43,44]. IL2 increased in severe AD, which secretes pruritus in almost all patients [45]. In addition, after treatment of the intranasal corticosteroid, the IL-2 were considerably diminished during 24 h, contained by significant enhancement in the upper airway function [43].

Collectively, this study indicates the potential ligands and critical targets to alleviate AM via orchestrating targets of AD, AR, and AA, providing theoretical clues for further experimental evaluation.

## 6. Conclusions

In summary, eight promising compounds (Bindarit, CTLA-4 inhibitor, Danirixin, A-205804, AX-24 HCl, Y-320, T-5224, and Apilimod) and eight targets (CCL2, CTLA4, CXCL8, ICAM1, IL10, IL17A, IL1B, and IL2) have been uncovered to alleviate AM with synergistic effects. Besides, the topological analysis on AM was orchestrated with a network-based concept. We conclude that multi-target agents are a potential strategy to treat comorbid AM, and network-based analysis especially provides critical clues to select targets on AM. Given the limitations of network pharmacology perspectives, the potential therapeutic ligands of the treatment of AM are predicted by topological analysis on PPI networks, and its efficacy in vivo was not validated, which needs to be further evaluated through therapeutic and clinical tests.

## Figures and Tables

**Table 1:** Degree of value of the top 30% by betweenness centrality (BC) in PPI.

No.	Target	Degree of Value
1	CCL2	19
2	CCL5	19
3	CSF2	19
4	CTLA4	19
5	CXCL8	19
6	FOXP3	19
7	ICAM1	19
8	IFNA1	19
9	IFNG	19
10	IL10	19
11	IL13	19
12	IL17A	19
13	IL1B	19
14	IL2	19
15	CCL20	18
16	CXCL10	18
17	CXCR4	18
18	IL18	18
19	CCR2	17
20	EGFR	15

**Table 2:** The physicochemical properties of 34 ligands related to 8 core targets.

No.	Compounds	Lipinski Rules				Lipinski’s Violations	Bioavailability Score	TPSA	Compound Classification
		MW	HBA	HBD	MLogP				
		<500	<10	≤5	≤4.15	≤1	>0.1	<140 Å^2^	
1	Bindarit	324.37	4	1	2.34	0	0.85	64.35	Organoheterocyclic compounds
2	Pirfenidone	185.22	1	0	2.49	0	0.55	22.00	Organoheterocyclic compounds
3	CTLA-4 inhibitor	427.35	7	1	4.61	1	0.55	68.50	Organoheterocyclic compounds
4	AZD5069	476.52	10	3	1.93	0	0.55	158.56	Organosulfur compounds
5	Danirixin	441.90	6	4	2.81	0	0.55	115.91	Benzenoids
6	A-205804	300.40	2	1	2.64	0	0.55	109.52	Organosulfur compounds
7	NECA	308.29	7	4	−2.6	0	0.55	148.41	Nucleosides, nucleotides, and analogues
8	Anemoside B4	1221.38	26	15	−4.58	3	0.17	412.82	Lipids and lipid-like molecules
9	AX-024 HCl	375.86	4	0	3.86	0	0.55	21.70	Phenylpropanoids and polyketides
10	Y-320	505.01	5	1	2.4	1	0.55	2.40	Benzenoids
11	A-740003	474.55	6	3	1.29	0	0.55	120.66	Benzenoids
12	Diacerein	368.29	8	1	1.14	0	0.55	124.04	Benzenoids
13	AUDA	392.58	3	3	3.95	0	0.55	78.43	Lipids and lipid-like molecules
14	o-Phenanthroline	180.21	2	0	1.86	0	0.55	25.78	Organoheterocyclic compounds
15	BC-1215	394.51	4	2	2.57	0	0.55	49.84	Organoheterocyclic compounds
16	3-Deazaadenosine hydrochloride	302.71	6	4	−1.78	0	0.55	126.65	Nucleosides, nucleotides, and analogues
17	GIBH-130	360.41	5	0	1.55	0	0.55	75.11	Organoheterocyclic compounds
18	Falcarindiol	260.37	2	2	3.33	0	0.55	40.16	Lipids and lipid-like molecules
19	Muscone	238.41	1	0	3.92	0	0.55	17.07	Organic oxygen compounds
20	T-5224	517.53	8	3	2.52	1	0.55	139.06	Benzenoids
21	Madecassic acid	504.70	6	5	3.33	1	0.55	118.22	Lipids and lipid-like molecules
22	RN-1734	353.31	4	1	2.89	0	0.55	57.79	Benzenoids
23	Stylopine	323.34	5	0	2.56	0	0.55	40.16	Alkaloids and derivatives
24	Andrographolide	350.45	5	3	1.98	0	0.55	86.99	Organoheterocyclic compounds
25	Dilmapimod	456.42	8	3	3.69	0	0.55	100.27	Organoheterocyclic compounds
26	Donepezil	379.49	4	0	3.06	0	0.55	38.77	Organoheterocyclic compounds
27	Etiprednol dicloacetate	485.40	6	1	3.08	0	0.55	89.90	Lipids and lipid-like molecules
28	Minocycline	457.48	8	5	−1.6	0	0.11	164.63	Phenylpropanoids and polyketides
29	Talmapimod	513.00	5	0	2.58	1	0.55	65.86	Organoheterocyclic compounds
30	VX-702	404.32	7	2	3.17	0	0.55	102.31	Organoheterocyclic compounds
31	VX-765	509.00	6	3	1.19	1	0.55	140.06	Organic acids and derivatives
32	Apilimod	418.49	6	1	1.69	0	0.55	84.76	Organic nitrogen compounds
33	RO2959 hydrochloride	463.93	6	1	2.96	0	0.55	99.25	Benzenoids
34	SU 5201	290.14	1	1	3.82	0	0.55	29.10	Organoheterocyclic compounds

**Table 3:** The binding energy of the eight targets related to eight ligands in the top 30% by betweenness centrality (BC).

				Grid Box		Hydrogen Bond Interactions	Hydrophobic Interactions
Protein	Ligand	PubChem ID	Binding Energy(kcal/mol)	Center	Dimension	Amino Acid Residue	Amino Acid Residue
CCL2 (PDB ID: 1DON)	Bindarit (★)	71354	−6.2	x = 61.620	x = 40	Asn6, Ala4	Pro2, Ile5, Ile51
				y = 0.356	y = 40		Ile20, Arg24, Lys49
				z = 0.582	z = 40		Asp3
	Pirfenidone	40362	−5.5	x = 61.620	x = 40	Thr16	Asn6, Arg18, Ile20
				y = 0.356	y = 40		Arg24, Lys49, Ile51
				z = 0.582	z = 40		Ala4
CTLA4 (PDB ID: 5XJ3)	CTLA-4 inhibitor (★)	101136468	−7.8	x = 22.471	x = 40	Ser32	His39, Tyr33, Phe53
				y = 17.954	y = 40		Ile43, Gly144
				z = −35.403	z = 40		
CXCL8 (PDB ID: 2IL8)	Danirixin (★)	24780598	−7.0	x = −0.977	x = 40	Asp52	Glu70, Ser72, Lys54
				y = −7.328	y = 40		Asn36, Cys34, Cys7
				z = 2.055	z = 40		Glu38, Cys9, Leu51
							Thr37, Thr12, Pro53
							Asn71
ICAM1 (PDB ID: 5MZA)	A-205804 (★)	9839311	−8.0	x = −5.082	x = 40	Gln998, Lys961	Asp911, Pro997, Pro1098
				y = −12.607	y = 40		Ala752, Gln755, Asp962
				z = 35.679	z = 40		Asp994, Tyr995, Ile996
IL10 (PDB ID: 1LK3)	AX-024 HCl (★)	129909862	−7.5	x = 17.574	x = 40	His41	Ser91,Thr116,Tyr181
				y = 49.569	y = 40		Leu114, Glu154, Gln39
				z = 27.605	z = 40		Asn93, Ala92
IL17A (PDB ID: 2VXS)	Y-320 (★)	22227931	−7.7	x = 77.528	x = 40	N/A	Gln93, Ser40, Glu95
				y = −4.484	y = 40		Pro63, Tyr62, Pro59
				z = −51.759	z = 40		Ser64, Val65, Pro37
							Trp67, Thr35, Gln94
	AX-024 HCl	129909862	−7.1	x = 77.528	x = 40	N/A	Gln93, Ser40, Asn36
				y = −4.484	y = 40		Pro63, Val65, Trp67
				z = −51.759	z = 40		Ile66, Ile96, Gln94
							Glu95, Pro37
IL1B (PDB ID: 1HIB)	T-5224 (★)	23626877	−8.0	x = 19.495	x = 40	Leu80, Val132, Gly136	Thr79, Tyr24, Glu25
				y = 2.991	y = 40	Thr137, Leu134	Phe133, Trp120, Gly135
				z = 73.516	z = 40		Lys77
	Dilmapimod	10297982	−7.7	x = 19.495	x = 40	Leu80,Leu26, Val132	Glu25, Phe133, Leu134
				y = 2.991	y = 40		Thr79, Tyr24, Gln81
				z = 73.516	z = 40		
	Stylopine	6770	−7.6	x = 19.495	x = 40	N/A	Ser125, Pro131, Val132
				y = 2.991	y = 40		Leu80, Glu25, Phe133
				z = 73.516	z = 40		
	Talmapimod	9871074	−7.4	x = 19.495	x = 40	Ser125, Thr79	Leu134, Glu25, Lys74
				y = 2.991	y = 40		Phe133, Pro131, Lys77
				z = 73.516	z = 40		
	A-740003	11351968	−7.3	x = 19.495	x = 40	Gly136	Thr137, Gly135, Asp142
				y = 2.991	y = 40		Phe133, Pro131, Lys77
				z = 73.516	z = 40		Thr79, Leu134, Trp120
	GIBH-130	50938773	−7.3	x = 19.495	x = 40	Ser43	Asn66, Leu62, Tyr68
				y = 2.991	y = 40		Leu6, Asn7, Ser5
				z = 73.516	z = 40		Pro91
	VX-702	10341154	−7.3	x = 19.495	x = 40	Ser43, Ser5, Glu64	Asn7, Pro91, Pro87
				y = 2.991	y = 40	Asn66	Tyr68, Tyr90, Lys63
				z = 73.516	z = 40		
	Madecassic acid	73412	−7.1	x = 19.495	x = 40	Leu80, Leu134, Lys77	Thr79, Phe133, Gly135
				y = 2.991	y = 40		Asp142, Thr137, Gly136
				z = 73.516	z = 40		
	Diacerein	26248	−7.0	x = 19.495	x = 40	Pro78, Thr79, Ser125	Pro131, Phe133, Gly135
				y = 2.991	y = 40		Lys77
				z = 73.516	z = 40		
	Donepezil	3152	−7.0	x = 19.495	x = 40	Ser125	Pro131, Met130, Phe133
				y = 2.991	y = 40		Thr79, Lys74, Lys77
				z = 73.516	z = 40		Leu134, Pro78
	Andrographolide	5318517	−6.8	x = 19.495	x = 40	Gly136, Thr137	Met130, Phe133, Pro131
				y = 2.991	y = 40		Trp120, Gly135, Asp142
				z = 73.516	z = 40		Ser125
	BC-1215	72201045	−6.7	x = 19.495	x = 40	Thr79	Pro131, Phe133, Ser125
				y = 2.991	y = 40		Asp142, Gly135, Leu134
				z = 73.516	z = 40		Lys77
	3-Deazaadenosine hydrochloride	134828261	−6.4	x = 19.495	x = 40	Asn66, Leu62, Glu64	Val85, Pro87, Gly61
				y = 2.991	y = 40		Ser43, Lys63, Tyr68
				z = 73.516	z = 40		Ser5, Pro91, Tyr90
	Etiprednol dicloacetate	9935073	−6.0	x = 19.495	x = 40	Thr79	Leu80, Tyr24, Leu134
				y = 2.991	y = 40		Lys77, Phe133, Glu25
				z = 73.516	z = 40		
	Muscone	10947	−5.9	x = 19.495	x = 40	N/A	Pro131, Phe133, Leu134
				y = 2.991	y = 40		Pro78, Thr79, Leu80
				z = 73.516	z = 40		
	AUDA	10069117	−5.7	x = 19.495	x = 40	Tyr90, Ser43, Pro87	Asn66, Leu62, Glu64
				y = 2.991	y = 40		Lys65, Lys63, Ser5
				z = 73.516	z = 40		Val3, Pro91, Tyr68
	o-Phenanthroline	1318	−5.5	x = 19.495	x = 40	Ser43	Gly61, Tyr68, Pro91
				y = 2.991	y = 40		Asn66, Leu62
				z = 73.516	z = 40		
	Falcarindiol	5281148	−5.0	x = 19.495	x = 40	Thr79, Glu25	Lys77, Pro78, Phe133
				y = 2.991	y = 40		Pro131, Leu80, Leu26
				z = 73.516	z = 40		Leu82, Val132
	RN-1734	3601086	−4.8	x = 19.495	x = 40	Glu64, Ser43	Tyr68, Leu62, Val85
				y = 2.991	y = 40		Pro87, Tyr90, Asn66
				z = 73.516	z = 40		Lys63
IL2 (PDB ID: 1M47)	Apilimod (★)	10173277	−6.2	x = 6.750	x = 40	Glu62, Lys43	Phe42, Pro65, Tyr107
				y = 25.891	y = 40		Tyr45
				z = 14.189	z = 40		
	RO2959 hydrochloride	45274292	−6.1	x = 6.750	x = 40	Asp20, His16	Asp84, Leu80, Leu12
				y = 25.891	y = 40		Gln13, Lys9, Asn88
				z = 14.189	z = 40		
	SU 5201	429070	−5.9	x = 6.750	x = 40	N/A	Glu62, Tyr107, Tyr45
				y = 25.891	y = 40		
				z = 14.189	z = 40		

(★): The most significant ligand on each target.

## Data Availability

All data generated or analyzed during this study are included in this published article (and its Supplementary Information files).

## Supplementary Materials

The following supporting information can be downloaded at https://www.mdpi.com/article/10.3390/cimb44050153/s1: Table S1: The number of 999 Atopic Dermatitis (AD) targets; The number of 517 Allergic Rhinitis (AR) targets; The number of 2374 Allergic Asthma (AA) targets, and The number of 229 Atopic March (AM) targets. Table S2: The DC-descending order of the number of 210 targets related to AM. Table S3: The top 30% DC-descending order of 65 targets extracted from Supplementary Table S2. Table S4: The top 30% BC-descending order of 20 targets extracted from Supplementary Table S3.

## Abbreviations

AD	Atopic Dermatitis
AM	Atopic March
AR	Allergic Rhinitis
AS	Asthma
BC	Betweenness Centrality
DC	Degree Centrality
MDT	Molecular Docking Test
IgE	Immunoglobulin E
PPI	Protein-Protein Interaction
